# Shall I Send My Boy to College?

**Published:** 1883-10

**Authors:** Ben. H. Pratt


### THE BISTOURY.

Vol. XX. XIX. ELMIRA, N. Y., OCTOBER, 1883. No. 3.

### Contributions.

### "SHALL I SEND MY BOY TO COLLEGE?"

BEN H. PRATT.

There are few parents, possessing the means of sending their boys through college, who have not seriously debated the propriety of doing so.

Parents are wont to have various reasons for sending their boys to college. One gives his boys a liberal education because he, the father, never had one, and thinks he would have been a very different and probably a much happier, or wealthier, or more famous man had he been given the same chance which he is now able to give his boy. Another wants his boy sent to college because he, the father, had a university education, and he wants to do as well by his son as he was done by. Another has ambitious designs, and hopes to see his son a somebody, a great preacher, or lawyer, or doctor, or professor, and feels that the youth should have a classical foundation upon which to build his career. Another feels that it is eminently respectable, quite the proper thing, for his boy to go through college, if only for the name of it. Another wants his son liberally educated, not for any special profession or calling in life; not because it is going to be especially beneficial to the young man in a pecuniary or business sense, but to round out his life more completely, and the better fit him to meet any of the emergencies of life, whatever they may be. Another sends his boy to college for that association with men of literary tastes which a college career entails, and for the culture which, aside from the knowledge he may acquire, is likely to result from such commingling. A few boys are sent to college because they desire to go, and often directly against the wishes of the parent. It would be impossible, in an article of this length, to state a tithe of the reasons which parents give for sending their boys through college. It would be equally impossible to recount the number of objections, good, bad and indifferent, to the placing of boys in college.

It is a great question, "Shall I send my boy to college?" I would not take the responsibility of answering the question for any one unless I well knew all the circumstances of the boy's life, his tastes, his bent, his capacity, his individuality.

There are hundreds of young men sent to college every year who never ought to have gone there; and there are scores prevented from going to college every year who will, their life through, bitterly regret that they were never given the opportunity to acquire liberal educations. Not all should go who want to go. Not all should be kept out of college because they don't want to go. It is a very difficult matter to decide whether a boy should be sent to college or into business.

Many circumstances combined must determine this question for the parent. The age of the boy is an important factor in the consideration. Ordinarily it takes four years to go through college. They are very valuable years to the lad. Somewhere, either in or out of college, he will probably pick up and store away within his mind during this particular four years more material than he will ever absorb in the same number of years afterward. He is of that age when that which comes in contact with him sticks to him and becomes a part of him. It is, therefore, most important to realize whether that which he will gather within himself at college will assimilate with that which he already has, and harmoniously enter into the mental composition of the youth, or whether it will be so diametrically at variance with the characteristics already possessed as to be in constant conflict therewith. These are points for careful consideration.

Disposition has much to do with the determination of a boy's educational career. There are boys who would seem to be in every particular just the persons to be sent to college, but for whom the college course, and the mingling with others which it implies, would be their worst possible environment. There are dispositions in which emulation stimulates all the germs of a broad manhood in a boy; and then there are those which cannot endure to be matched with others, but must be developed apart. Emulation makes the one and mars the other. Discretion is a chief consideration. Unless a boy is pretty well balanced, and has a good deal of moral stamina, the associations of college life will unquestionably do him more harm than good. Parents that have never gone through with a college course cannot realize what an ordeal it is. The best colleges in the land are the grandest aggregations of deviltry that can be found. The brighter the intellects that are gathered the more brilliant will the concentrated vice there be. It has never been satisfactorily explained why it is that our colleges should be hot-beds of vice and immorality, but such is the undisputed fact. Has the young man whom you would place among these brilliant imps of Satan the necessary moral momentum to carry him through a four-years mingling with them?

Again, is the youth whom you would send to college one who, having lived four years in an artificial world, apart from all the practicalities of life, breathing a morally and intellectually factitious atmosphere, and one wholly ideal in its character, able to abandon, at the close of the college term, all that he has there found, and take a place among the men of a wholly different sort, whom he must necessarily meet as soon as he leaves the halls of learning? Can he be so thoroughly impressed, prior to his entrance into this artificial world, with a true sense of its artificiality, and carry with him a realizing sense that what he finds in college is in no sense what he will find after leaving it? Is it likely to unfit him for life's duties? This is probably one of the greatest dangers that compass the career of every young man that is sent to college. He forms a wrong estimate of men from being associated with a class which forms so small a portion of the world in which he is eventually to buffet for a livelihood, that unless he fully and continually realizes that the one is artificial and temporary and the other almost the very antipodes of it, he will get ideas that will make him miserable for the greater portion of his life, and mar his success beyond computation.

It is almost impossible to find, among those young men destined for college, one in ten that has the necessary qualifications to enter and accomplish a college course and come forth in every respect as good a man and as well qualified for the duties of life as he would have been by devoting the time and labor he expended while in college to the acquirement of a knowledge of the business which he purposed to pursue. I do not put this upon mercenary grounds. I would not have the youth remain out of college because he can make more money than by going to college, but I do claim that while the one is being drilled constantly in that which he is to do, the other is being kept in startling ignorance of what he must eventually meet, and for which he will find himself wholly unprepared.

The trouble is not that so much time is expended in securing a college education that the youth may not recover it for the practical uses of after life, but that the association is such that he cannot, for many years after his college days are over, step out of the peculiar surroundings he has become wonted to, and into those which meet him when he has passed out forever from the doors of his alma mater. The young man, as a rule, goes into college in a mentally plastic state, and is there moulded in accordance with what he there meets. He finds young men chiefly of his own class, intellectually and socially. The tendency of the college curriculum is to develop the intellectual and social abnormally, as compared with the average of youth. The boy naturally drifts away from the actual and dwells with the ideal. He is not brought into contact with facts, but with the images or shadows of facts. Facts are hard. They rub, they chafe, they hit, they hurt, and he who meets them, learns to encounter them, to handle them, to buffet them, to endure them, to fight them, to use them. The ideal is shape, not substance. It does not impinge and leave either pain or scar. One but plays fight when he combats them. One's whole college career consists of setting up imaginary foes and always vanquishing them. One is drawn inferentially to the conclusion that his experience within college is what he may expect beyond it, and he emerges from his ideal world with distorted views of well nigh everything material. It takes years of mingling with men in the real world to disabuse his mind of its misconceptions. He finds that only one out of a hundred knows from what language he quotes a phrase commonplace within classic halls; and but one of five hundred, perhaps, who recognizes the author and the interpretation of the phrase. He is continually shocked at the seeming ignorance of his kind, and his friends are equally surprised, but more amusedly, at his pedanticism. It takes him years to realize how little he has been taught of what he must know, and how much of what he begins to feel that he never will have use for. The grand diploma, for which he labored hard during four years, and which he has set such store by as the passport to immediate and proud success in life, becomes less and less prized as the years roll on, and he finally packs it away with his Calculus and his Juvenal, and his Sophocles and his belle-lettres, and forgets that any of them ever were. He has had to learn much only to unlearn it, because there is no market for his acquirements in the work-a-day world that he must rough and tumble in. Alas, for the young fellow that comes out of college and thinks himself prepared for the business of life. He may find a congenial professorship to squat into and struggle with, or he may have a taste for one of the professions and find his declensions and his equations and his syllogisms now and then of some use, but even then he realizes how much defunct matter he has been for years resurrecting, only to find it impalpable dust.

By the casual observer, it is naturally surmised that a college education, if attainable without very great sacrifice, cannot be a disadvantage to any, and must be an advantage to most. So it would be if our colleges were more practical in their *modus operandi.* But, with all the progressiveness that has characterized this age, it is a remarkable fact that the college course of to-day—the classical course which most seek to accomplish—is not in any material point different from that of a half century ago. The same stress is laid upon the acquirement of the dead languages; upon the abstruse in mathematics; upon the abnormally metaphysical that was demanded half a century since. With all the young men that are annually poured out upon their several communities, and found floating hither and yon in a dazed sort of way, wondering why something doesn't turn up for them, and speculating upon the malarrangement of mundane affairs that doesn't afford them a place among men, it ought to be discovered that there's something wrong in the process that has unfitted all these men for the hurly-burly of active business life. While these young gentlemen have been soaring amid the higher strata of educational achievement, the boys who went out of the academies and high schools have stepped into business and are fairly along with the mastery of all its details, and the college man seems to find no place to enter the ranks unless he goes back to where he left off to enter college and counts his four years of study—for the present—as a nullity. Few are willing to do this. Few are able to realize that their college-gained knowledge is something for the future, and not for the nonce. If they could do this readily, and begin at the close of a college course just where they would have begun at the time of entering college, the mere loss of four years would be trifling, and be more than compensated for in the self-satisfaction which they would possess in the consciousness of having much which the non-graduate cannot have, and does not realize the worth of.

Here, then, is the one point for all young men to carefully note: If they can go to and through college and come out prepared to take just such a place as they would have accepted before entering college, then college will not spoil them nor mar their business prospects one whit, but rather make of them more complete men, broader men, better citizens and far more useful adjuncts to society. If any father thinks that by his and his son's combined effort the latter can go to college and be reconciled to a business start four years back of the fellows of his own age, then the question, "Shall I send my boy to college?" may be safely answered in the affirmative. Otherwise, don't send your boy to college.

